# Knowledge and Awareness of Anaphylaxis and Anaphylactic Shock Among the General Population of the Western Region of Saudi Arabia

**DOI:** 10.7759/cureus.72683

**Published:** 2024-10-30

**Authors:** Ali Almontasheri, Adeeb Munshi, Shomokh F Alotaibi, Areej Munshi, Ali Alghamdi, Salman A Hakami

**Affiliations:** 1 Adult Allergy and Clinical Immunology, King Abdulaziz Medical City, Jeddah, SAU; 2 Internal Medicine, King Abdulaziz Medical City, Jeddah, SAU; 3 Family Medicine, King Abdulaziz Medical City, Jeddah, SAU; 4 Dermatology, King Abdulaziz Medical City, Jeddah, SAU; 5 Psychiatry, Mental Health Hospital, Ministry of Health, Jeddah, SAU

**Keywords:** allergy, anaphylaxis, awareness, knowledge, shock

## Abstract

Introduction

Anaphylactic shock is a severe and acute life-threatening condition triggered by exposure to common allergens. Despite the increasing incidence of anaphylaxis worldwide, there is still a lack of awareness and understanding of anaphylaxis and its proper management.

Methods

A descriptive survey design was used to evaluate the knowledge and awareness of anaphylactic shock among the general population in the western region of Saudi Arabia through an online questionnaire distributed from January 2024 to June 2024. The validated questionnaire included demographic information and 18 knowledge-based questions on anaphylaxis.

Results

The data was collected from 501 participants in the western region of Saudi Arabia. Among them, 290 (58%) were female, and 211 (42%) were male. The largest age group was between 41 and 50 years, comprising 121 participants (24.1%). Additionally, most participants, 298 (59.4%), held a bachelor's degree. A total of 207 participants (41.3%) reported either experiencing anaphylaxis or knowing someone who had. Furthermore, 123 participants (24.5%) underwent tests for anaphylaxis or anaphylactic shock, with 88 (17.5%) having a blood test and 35 (7%) having a skin prick test. In terms of knowledge about anaphylaxis, 266 participants (53%) were able to accurately identify multiple symptoms, while 195 (38.9%) were uncertain about the proper diagnostic methods. Moreover, only 92 (18.4%) recognized epinephrine as the first-line treatment, and 125 (25%) knew the correct administration route (intramuscular). 216 participants (43.1%) were aware of the need to visit the ER after using epinephrine. Furthermore, the survey findings indicated that 318 participants (63.5%) acknowledged the dangerous nature of anaphylaxis, with 258 (51.5%) believing that it could be fatal. However, 228 (45.5%) remained unsure about the risk of anaphylaxis-associated organ failure.

Conclusion

The study highlighted the need for targeted public awareness campaigns to improve understanding and ensure early recognition and correct management of anaphylaxis.

## Introduction

Anaphylactic shock is a critical and potentially fatal systemic reaction that can manifest within seconds or minutes following exposure to an allergen, such as food, medication, or an insect sting. These allergens influence the release of immunological mediators, such as immunoglobin E (IgE), followed by histamine release or prostaglandins [1]. These mediators will cause the clinical manifestations of anaphylaxis, including bronchoconstriction, vasodilation, hypotension, tachycardia, dyspnea, flushing, urticaria, and angioedema [2]. Food, medications, and stinging insects are major triggers for anaphylaxis. Idiopathic anaphylaxis occurs more commonly in adults (30%-60%) than in pediatrics (10%) [3]. The first-line therapy for anaphylactic shock is intramuscular (IM) epinephrine injection. This medication widens the blood vessels and airways, raises blood pressure, and reduces angioedema. Consequently, it effectively alleviates the symptoms of anaphylaxis [4]. In the United States, anaphylaxis occurs annually in 30 per 100,000 people, with a reported mortality of 1-2% [5]. A second cross-sectional study conducted in Saudi Arabia indicated that the prevalence of anaphylaxis among emergency department (ED) admissions was 0.00026%. Among these cases, 98 (60.9%) were pediatric (ages 1-16 years), and 63 (39.1%) were adults (ages 17-40 years) [6]. Moreover, in recent studies, the incidence and prevalence of anaphylaxis have been increasing over the last two decades [7, 8].

Anaphylaxis is universal and can occur in people of all age groups. In Australia, the incidence was found to be equal in adults and children, but in the Middle East, the prevalence was reported to be high in children and critical in adults [6, 9]. Anaphylaxis can cause cardiac and respiratory arrest and lead to death within minutes, so prompt assessment and treatment of anaphylaxis is crucial [10]. Although the management of epinephrine is life-saving, it is underused by people to treat anaphylactic reactions. People avoid administering the epinephrine IM injection for lack of availability, safety concerns, incorrect administration, and injury [4]. In Saudi Arabia, a low level of awareness and assessment was reported [8].

A cross-sectional study was conducted among school teachers at a public school in Al-Qassim, Saudi Arabia, and it was determined that teachers need better overall knowledge and practice regarding anaphylactic shock. The study found that out of 384 Saudi school teachers, 85.3% had a low level of knowledge, 13.7% had moderate knowledge, and only five had a high level of knowledge. In terms of practice, 47.5% reported low levels, 48.9% reported moderate levels, and 3.6% reported high levels [8]. A second cross-sectional study was conducted in Al Taif, Saudi Arabia. It revealed a lack of knowledge and awareness of anaphylactic shock and its management among mothers of medically diagnosed children with food allergies [11]. It is unclear whether people know the signs, symptoms, and complications of anaphylaxis and how to manage it [7, 9]. Therefore, this study aims to assess the knowledge and awareness of anaphylaxis and anaphylactic shock among the public of the western region of Saudi Arabia. This will assist in reducing the incidence of morbidity and mortality and promoting general public awareness and action of anaphylactic shock.

## Materials and methods

Study design

The study employed a descriptive survey design. Researchers can utilize correlation survey data to forecast future trends and elucidate the characteristics of a population or variances among the general population in the western region of Saudi Arabia. Furthermore, this design's reliability in maintaining respondents' anonymity, which encourages them to provide honest responses, makes it well-suited for this study.

Study population

The questionnaire was distributed among the general population in the western region of Saudi Arabia. All respondents were consented before completing the questionnaire. The purpose and background of the study were added to the first page of the questionnaire. Participants were free to withdraw from the study at any time, even without providing a reason, and their opinions and information would be anonymous.Study instrument

The data were collected through an online, closed-ended questions questionnaire distributed over a six-month period from January 2024 to June 2024. The survey was designed to encompass the general populace of the western region of Saudi Arabia. The questionnaire survey was validated and modified based on previous studies [7, 10, 11]. Additionally, two allergy and immunology experts and one family medicine expert reviewed the questionnaire for validity.

The first part of the questionnaire, crucial for understanding the participants' backgrounds, was designed to gather important demographic information (gender, age, nationality, education level, marital status, employment status, monthly income, and chronic disease). This was followed by the second part, which included 18 questions to assess participants' knowledge of anaphylaxis or anaphylactic shock. The validity of the questionnaire was evaluated through a pilot study, while its reliability and internal consistency were assessed using Cronbach's alpha.

Data collection

Participants were enlisted through a chain-referral sampling method. The questionnaire was disseminated via links on various platforms, such as Twitter and WhatsApp. Participants were encouraged to distribute the questionnaire among their family, friends, and colleagues.

Statistical analysis

The data was entered into Microsoft Excel (MS Office, 2010) for updating and later analyzed using SPSS software version 22.0 (SPSS Inc., Chicago, Ill). The analysis included computing frequencies and percentages for descriptive statistics. Numbers represented categorical variables, while mean ± standard deviation (SD) was used to describe quantitative variables. We utilized the Shapiro-Wilk test to evaluate whether the variable distribution followed a normal pattern. Parametric variables between study groups were compared using a one-way analysis, and non-parametric variables were compared using the Kruskal-Wallis test. Qualitative variables were assessed using the chi-square test, with a p-value of less than 0.05 considered to indicate statistical significance.

Ethical consideration

The study was approved by the Institutional Review Board of King Abdullah International Medical Research Center with the approval number NRJ21J/158/06.

## Results

The study included 501 participants from the western region of Saudi Arabia. The majority of respondents were female (290 participants, 58%), and most were Saudi nationals (488 participants, 97.4%). The participants were predominantly well-educated, with a majority holding a bachelor's degree (298 participants, 59.4%). The majority of participants were married (357 participants, 71.3%) and employed (253 participants, 50.5%). In terms of income, the largest group reported earning less than 5,000 SR (159 participants, 31.7%). Most participants (381 participants, 76%) reported no chronic diseases, although there were significant proportions reporting diabetes mellitus (60 participants, 12%) and hypertension (74 participants, 14.8%) (Table 1).

**Table 1:** Demographics information of the participants * The participant can choose more than one chronic disease. The data has been represented as N, %. DM: Diabetes mellitus, HTN: Hypertension

Variable	Number	Percentage
Gender		
Male	211	42%
Female	290	58%
Age		
From 18-20 years	28	5.6%
From 21-30 years	103	20.5%
From 31-40 years	108	21.5%
From 41-50 years	121	24.1%
From 51-60 years	105	20.9%
61 years and above	36	7.1%
Nationality		
Saudi	488	97.4%
Non-Saudi	13	2.6%
Education level		
Primary school	1	0.2%
Intermediate school	7	1.4%
Secondary school	98	19.5%
Diploma	40	8%
Bachelor	298	59.4%
Master	39	7.8%
PhD	18	3.6%
Marital status		
Single	114	22.7%
Married	357	71.3%
Divorced	20	4%
Widow	10	2%
Employment status		
Student	80	16%
Employed	253	50.5%
Unemployed	86	17.1%
Retired	82	16.4%
Monthly income		
Less than 5000 SR	159	31.7%
From 5000-10000 SR	99	19.8%
From 10000-15000 SR	78	15.6%
From 15000-25000 SR	120	24%
More than 25000 SR	45	9%
Chronic diseases*		
DM	60	12%
HTN	74	14.8%
Cardiac disease	22	4.4%
Respiratory disease	33	6.6%
Non	381	76%

**Figure 1:**
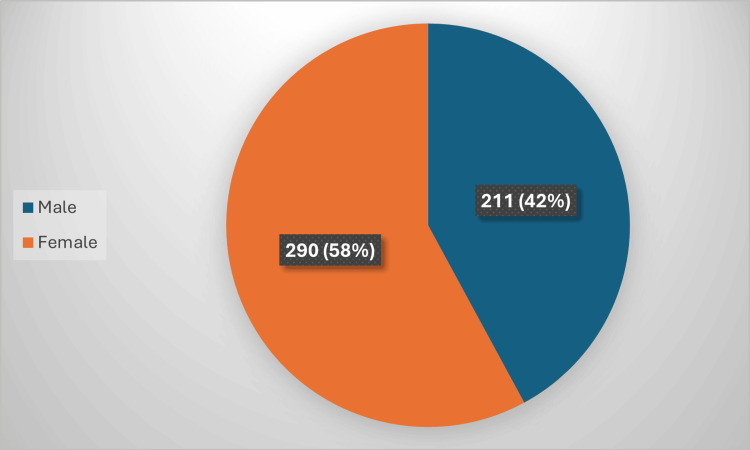
Gender distribution of the participants The data has been represented as N, %.

Figure 1 shows the gender distribution among the participants. Of the 501 respondents, 290 (58%) identified as female, while 211 (42%) identified as male.

**Figure 2:**
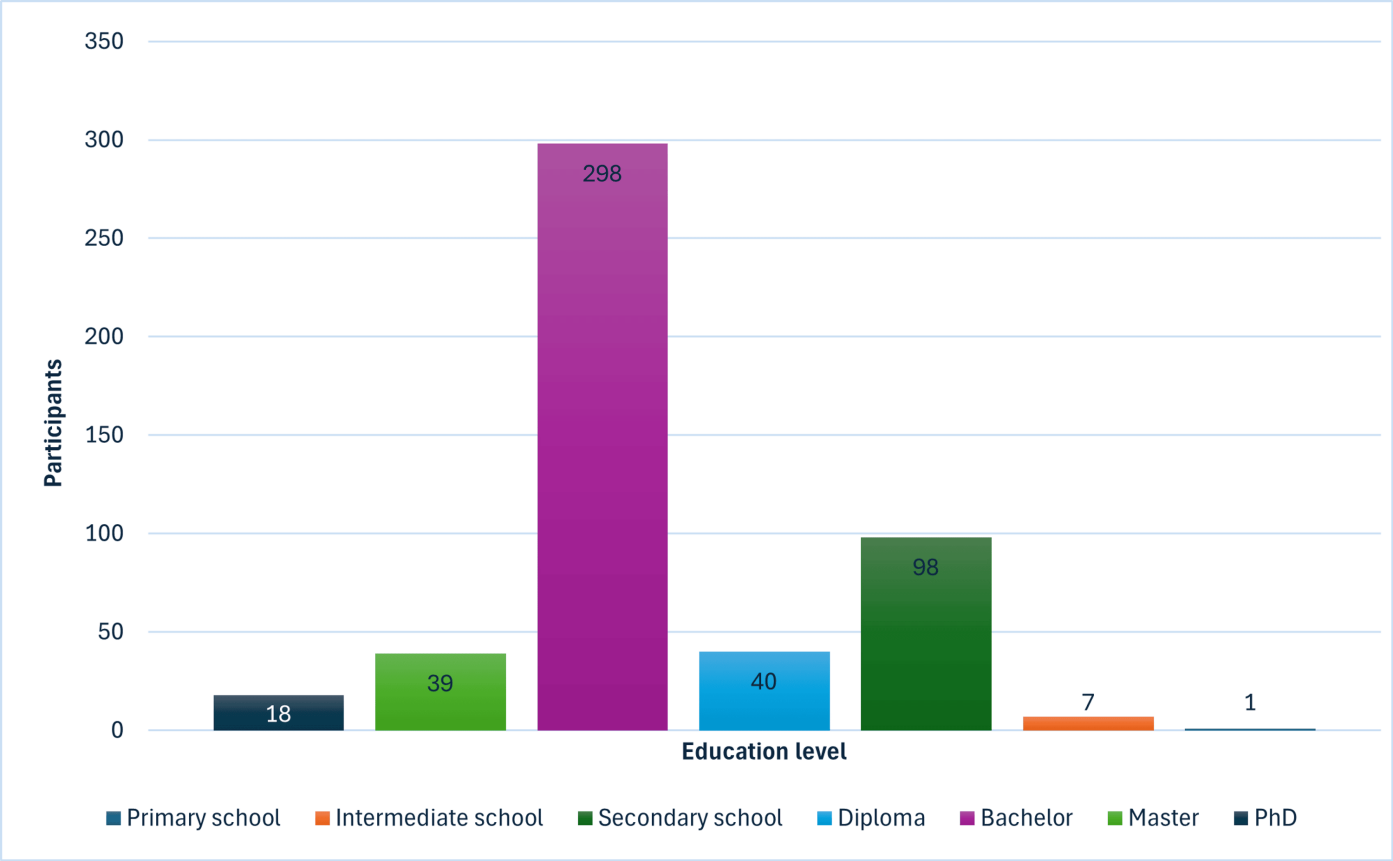
The education level of the participants The data has been represented as N, %.

The data presented in Figure 2 illustrates the educational level of the participants. It indicates that the majority of the sample has a high level of education. The largest proportion have obtained a bachelor's degree, constituting 298 (59.4%) of the individuals. Following this, 39 (7.8%) participants hold a master's degree, and 18 (3.6%) individuals have completed a PhD. Additionally, there are 98 (19.5%) participants with a Secondary School education and 40 (8%) with a Diploma. A small number of participants, 7 (1.4%), have completed Intermediate School, and only one (0.2%) has completed Primary School.

The questionnaire revealed that 207 participants (41.3%) either experienced anaphylaxis or knew someone who did. Out of the participants, 123 (24.5%) underwent tests for anaphylaxis or anaphylactic shock, with 88 (17.5%) having a blood test and 35 (7%) having a skin prick test. While 266 (53%) participants accurately identified multiple symptoms of anaphylaxis, 195 (38.9%) were uncertain about the proper diagnostic methods for anaphylaxis. In terms of preventive measures, 209 (41.7%) participants recognized the importance of avoiding triggers and using corticosteroids or antihistamine tablets. Only 92 (18.4%) participants identified epinephrine as the first-line treatment, and just 125 (25%) knew the correct administration route (intramuscular). Furthermore, only 216 (43.1%) were aware of the need to visit the ER after epinephrine use. It was acknowledged by 318 (63.5%) participants that anaphylaxis is a dangerous condition, and 258 (51.5%) believed that it could be fatal. However, 228 (45.5%) remained unsure of the risk of organ failure associated with anaphylaxis. Additionally, 232 (46.3%) participants mistakenly believed that anaphylaxis or anaphylactic shock is a hereditary condition, and 312 (62.3%) also mistakenly believed that it is a chronic condition. On the bright side, 346 (69%) participants correctly believed that anaphylaxis or anaphylactic shock is not infectious. The questionnaire's participants identified food as the most common cause of anaphylaxis and anaphylactic shock, with 321 (64.1%) mentioning it, followed by drugs with 248 (49.5%). Nuts and peanuts were identified as the most common food allergen, with 304 (60.7%) participants mentioning it, followed by eggs at 296 (59%) and seafood at 282 (56.3%) (Table 2).

**Table 2:** Knowledge and awareness of the participants towards anaphylaxis and anaphylactic shock * The participant can choose more than one answer. The data has been represented as N, %. EpiPen: Epinephrine autoinjector

Variables	Number	Percentage
Have you or anyone of your friends/family suffered or currently suffering from anaphylaxis or anaphylactic shock?		
Yes	207	41.3%
No	294	58.7%
If yes, what did you/they do?		
Went to the ER	91	18%
I used EpiPen injection	9	1.8%
Anti-histamine medication	89	17.8%
Nothing	18	3.7%
I never suffered nor any one from my friends or family from anaphylaxis or anaphylactic shock	294	58.7%
Have you ever been worked-up for anaphylaxis or anaphylactic shock?		
Yes	123	24.5%
No	378	75.5%
Which tests have been performed on you?		
Blood test	88	17.5%
Skin prick test	35	7%
I have never been worked-up for anaphylaxis or anaphylactic shock	378	75.5%
Which one of the following can anaphylaxis present with?		
Respiratory symptoms (dyspnea, wheeze, stridor, hypoxemia, inability to maintain patency, and persistent cough)	93	18.5%
Hypotension	2	0.4%
Skin manifestations (hives, rash, erythema, pruritus, swelling of the lips)	129	25.7%
Gastrointestinal symptoms (stomachache, nausea, vomiting, and diarrhea)	11	2.2%
All of the above	266	53%
What is the most proper method to confirm anaphylaxis or anaphylactic shock diagnosis?		
Blood test	219	43.7%
Skin prick test	87	17.4%
I don't know	195	38.9%
What do you think is the proper way to prevent anaphylaxis or anaphylactic shock?		
Avoidance of the triggers	198	39.5%
Corticosteroids or anti-histamine tablets	8	1.6%
All of the above	209	41.7%
I don't know	86	17.2%
What do you think is the cornerstone treatment for anaphylaxis or anaphylactic shock?		
Anti-histamine	169	33.7%
Corticosteroid tablets	33	6.6%
EpiPen	92	18.4%
I don't know	207	41.3%
What do you think is the route of epinephrine injection?		
Intramuscular	125	25%
Subcutaneous	41	8.2%
Intravenous	45	9%
Orally	13	2.6%
I don't know	277	55.3%
What do you think you should do after injecting the EpiPen?		
Nothing	63	12.6%
Go to the ER	216	43.1%
I don't know	222	44.3%
Do you think anaphylaxis or anaphylactic shock is a dangerous condition for your health?		
Yes	318	63.5%
No	47	9.4%
I don't know	136	27.1%
Do you think anaphylaxis or anaphylactic shock can cause organ failure?		
Yes	191	38%
No	82	16.4%
I don't know	228	45.5%
Do you think anaphylaxis or anaphylactic shock can lead to death?		
Yes	258	51.5%
No	60	12%
I don't know	183	36.5%
Do you think anaphylaxis or anaphylactic shock is a hereditary condition?		
Yes	232	46.3%
No	84	16.8%
I don't know	185	36.9%
Do you think anaphylaxis or anaphylactic shock is an infectious condition?		
Yes	46	9.2%
No	346	69%
I don't know	109	21.8%
Do you think anaphylaxis or anaphylactic shock is a chronic disease?		
Yes	312	62.3%
No	60	12%
I don't know	129	25.7%
What do you think can cause anaphylaxis or anaphylactic shock?*		
Drugs	248	49.5%
Food	321	64.1%
Insect sting	181	36.1%
Latex	91	18.2%
I don't know	132	26.3%
What do you think are the common foods that can cause anaphylaxis or anaphylactic shock?*		
Eggs	296	59%
Nuts- peanuts	304	60.7%
Milk	185	37%
Seafood	282	56.3%
I don't know	92	18.4%

The results of the questionnaire revealed significant gender differences in understanding anaphylaxis. 57 (19.7%) females correctly identified that epinephrine injection is the primary treatment for anaphylaxis or anaphylactic shock, compared to 35 (16.6%) males (p=0.045). When asked about the proper route of epinephrine injection, 124 (58.8%) males and 153 (52.8%) females admitted they didn't know, with only 56 (26.5%) males and 69 (23.8%) females correctly identifying the intramuscular route (p = 0.022). Moreover, when asked whether anaphylaxis could be an infectious condition, 32 (15.2%) males and 14 (4.8%) females incorrectly answered "yes," while the majority, 177 (55.5%) males and 229 (79%) females, correctly answered "no." This resulted in a highly significant p-value of <0.001, indicating a substantial gap in awareness between the genders. Additionally, regarding the potential severity of anaphylaxis, 162 (55.9%) females believed it could lead to death, compared to 96 (45.5%) males, resulting in a significant p-value of 0.019. Furthermore, 195 (67.2%) females recognized nuts and peanuts as common causes of anaphylaxis, compared to 109 (51.7%) males (p < 0.001), indicating that females were more knowledgeable about this food trigger. These findings underscore the need for better public awareness and education, especially on critical life-saving interventions such as the correct use of epinephrine and recognizing food triggers (Table 3).

**Table 3:** Comparison of knowledge and awareness of anaphylaxis and anaphylactic shock between males and females EpiPen: epinephrine autoinjector The data has been represented as N, %. A p-value of less than 0.05 is considered to indicate statistical significance

Question	Answer	Male		Female		Pearson Chi-Square	p-value
		N	%	N	%		
Have you or anyone of your friends/family suffered or currently suffering from anaphylaxis or anaphylactic shock?	Yes	83	39.3%	124	42.8%	0.59	0.442
	No	128	60.7%	166	57.2%		
If yes, what did you/they do?	I never suffered nor any one from my friends or family from anaphylxis or anaphylactic shock	129	61.1%	166	57.2%	3.767	0.438
	Anti-histamine medication	32	15.2%	57	19.7%		
	Went to the ER	36	17.1%	55	19.0%		
	I used EpiPen injection	5	2.4%	3	1.0%		
	Nothing	9	4.3%	9	3.1%		
Have you ever been worked-up for anaphylaxis or anaphylactic shock?	Yes	54	25.6%	69	23.8%	0.213	0.644
	No	157	74.4%	221	76.2%		
Which tests have been performed on you?	Blood test	38	18.0%	50	17.2%	0.279	0.644
	Skin prick test	16	7.6%	19	6.6%		
	I have never been diagnosed with anaphylaxis or anaphylactic shock	157	74.4%	221	76.2%		
Which one of the following can anaphylaxis present with?	Respiratory symptoms	34	16.1%	59	20.3%	6.986	0.137
	Hypotension	0	0.0%	2	0.7%		
	Skin manifestations	47	22.3%	82	28.3%		
	Gastrointestinal symptoms	5	2.4%	6	2.1%		
	All of the above	125	59.2%	141	48.6%		
What is the most proper method to confirm anaphylaxis or anaphylactic shock diagnosis?	Blood test	82	38.9%	137	47.2%	4.019	0.134
	Skin prick test	37	17.5%	50	17.2%		
	I don't know	92	43.6%	103	35.5%		
What do you think is the proper way to prevent anaphylaxis or anaphylactic shock?	Avoidance of the triggers	84	39.8%	114	39.3%	3.238	0.356
	Corticosteroids or anti histamine tablets	3	1.4%	5	1.7%		
	All of the above	81	38.4%	128	44.1%		
	I don't know	43	20.4%	43	14.8%		
What do you think is the cornerstone treatment for anaphylaxis or anaphylactic shock?	Antihistamine	66	31.3%	103	35.5%	8.043	0.045
	Corticosteroid tablets	9	4.3%	24	8.3%		
	Epinephrine injection	35	16.6%	57	19.7%		
	I don't know	101	47.9%	106	36.6%		
What do you think is the route of epinephrine injection?	Intramuscular	56	26.5%	69	23.8%	11.444	0.022
	Subcutaneous	15	7.1%	26	9.0%		
	Intravenous	9	4.3%	36	12.4%		
	Orally	7	3.3%	6	2.1%		
	I don't know	124	58.8%	153	52.8%		
What do you think you should do after injecting the EpiPen?	Nothing	26	12.3%	37	12.8%	0.413	0.814
	Go to the ER	88	41.7%	128	44.1%		
	I don't know	97	46.0%	125	43.1%		
Do you think anaphylaxis or anaphylactic shock is a dangerous condition for your health?	Yes	121	57.3%	197	67.9%	8.975	0.011
	No	18	8.5%	29	10.0%		
	I don't know	72	34.1%	64	22.1%		
Do you think anaphylaxis or anaphylactic shock can cause organ failure?	Yes	74	35.1%	117	40.3%	3.307	0.191
	No	31	14.7%	51	17.6%		
	I don't know	106	50.2%	122	42.1%		
Do you think anaphylaxis or anaphylactic shock can lead to death?	Yes	96	45.5%	162	55.9%	7.895	0.019
	No	23	10.9%	37	12.8%		
	I don't know	92	43.6%	91	31.4%		
Do you think anaphylaxis or anaphylactic shock is a hereditary condition?	Yes	88	41.7%	144	49.7%	3.284	0.194
	No	40	19.0%	44	15.2%		
	I don't know	83	39.3%	102	35.2%		
Do you think anaphylaxis or anaphylactic shock is an infectious condition?	Yes	32	15.2%	14	4.8%	33.744	<0.001
	No	117	55.5%	229	79.0%		
	I don't know	62	29.4%	47	16.2%		
Do you think anaphylaxis or anaphylactic shock is a chronic disease?	Yes	118	55.9%	194	66.9%	6.897	0.032
	No	27	12.8%	33	11.4%		
	I don't know	66	31.3%	63	21.7%		
What do you think can cause anaphylaxis or anaphylactic shock?	Drugs	111	52.6%	137	47.2%	1.406	0.236
	Food	123	58.3%	198	68.3%	5.287	0.021
	Insect sting	66	31.3%	115	39.7%	3.713	0.054
	Latex	41	19.4%	50	17.2%	0.394	0.53
	I don't Know	62	29.4%	70	24.1%	1.732	0.188
What do you think are the common food that can cause anaphylaxis or anaphylactic shock?	Eggs	132	62.6%	164	56.6%	1.823	0.177
	Nuts- peanuts	109	51.7%	195	67.2%	12.43	<0.001
	Milk	82	38.9%	103	35.5%	0.587	0.444
	Seafood	116	55.0%	166	57.2%	0.255	0.614
	I don't know	47	22.3%	45	15.5%	3.72	0.054

## Discussion

The present study aims to assess the knowledge and awareness regarding anaphylaxis and anaphylactic shock in the western region of Saudi Arabia. The results revealed misconceptions regarding this life-threatening condition, including the diagnosis, symptoms, and management. Although many of the population has some exposure to anaphylaxis, knowledge gaps remain.

Anaphylaxis is becoming more common worldwide, with many studies reporting high prevalence rates [3, 12]. This increase is often linked to greater exposure to common allergens like food, insect stings, medications, and latex [9, 13]. Our study revealed a relatively high level of exposure, with 207 participants (41.3%) either having personally experienced anaphylaxis or knowing someone who has. This may be attributed to a general misunderstanding of what anaphylaxis is, as many people misuse the term by referring to allergic reactions in general. Surprisingly, only 123 (24.5%) participants had been evaluated for anaphylaxis previously. This suggests that many cases of anaphylaxis may go undiagnosed, which is concerning as failing to recognize the condition can lead to improper treatment and an increased risk of severe complications [14].

Early recognition of anaphylaxis symptoms is crucial, as it will aid in effective treatment [15]. While 266 (53%) participants in our study could identify a range of symptoms, including respiratory, cardiovascular, skin, and gastrointestinal manifestations, many focused primarily on visible or commonly known symptoms, such as skin reactions 129 (25.7%) and difficulty breathing 93 (18.5%). Surprisingly, only 2 (0.4%) participants recognized hypotension as a symptom, even though cardiovascular collapse is one of the most dangerous aspects of anaphylaxis [16]. This suggests that while some participants know the diversity of symptoms, others focus on the more visible or commonly mentioned manifestations. This lack of awareness highlights the need to improve education on the various symptoms of anaphylaxis to minimize the risk of delayed treatment and serious complications.

Anaphylaxis is a severe emergency condition that involves the whole body and is primarily diagnosed based on clinical symptoms. The diagnosis is made based on the specific symptoms that occur shortly after exposure to a known or suspected allergen [17]. While skin prick testing is commonly used to identify specific IgE-mediated allergies, it is not typically used to diagnose anaphylaxis [18]. On the other hand, serum tryptase, a biomarker released by mast cells during an anaphylactic reaction, can help in diagnosing anaphylaxis [19]. When measured within the first two hours of symptom onset, the tryptase level has shown to be a sensitive diagnostic marker for anaphylaxis [20], with higher levels correlating with the severity of the reaction [21]. Additionally, follow-up testing is recommended to establish a baseline and to differentiate anaphylaxis from other allergic reactions [22]. Our study found that 219 (43.7%) participants correctly identified blood tests as the appropriate method for confirming an anaphylaxis diagnosis, while 195 (38.9%) were unsure, indicating a misunderstanding regarding the diagnosis of anaphylaxis.

In our study, we found that there was a partial understanding of preventive measures for anaphylaxis. While 209 (41.7%) participants correctly identified avoiding triggers and using antihistamines or corticosteroids as effective prevention strategies, 198 (39.5%) believed that avoiding triggers alone was sufficient. In addition to avoiding known allergens, individuals at high risk for anaphylaxis should also be informed about the importance of carrying an epinephrine autoinjector (EpiPen) and other essential medications [3].

The study results regarding treatment showed that only 92 (18.4%) participants correctly identified epinephrine as the primary treatment for anaphylaxis. Despite the widespread recognition of epinephrine as the cornerstone treatment for anaphylaxis [23], 169 (33.7%) believed that antihistamines were the primary therapy, and a smaller group, 33 (6.6%), thought that corticosteroid tablets were the key treatment. These beliefs reflect a dangerous misunderstanding that could lead to improper management of anaphylaxis, as antihistamines are not sufficient to reverse the life-threatening symptoms of anaphylaxis, particularly cardiovascular and respiratory symptoms [24]. These results are consistent with other studies that show a tendency to overestimate the efficacy of antihistamines and delay the use of epinephrine in treating anaphylaxis [25]. Results also showed confusion regarding the proper route of epinephrine administration; only 125 (25%) participants knew that the intramuscular route was the preferred method. Many individuals, 277 (55.3%), were unsure, and others incorrectly identified intravenous, subcutaneous, or even oral routes, which could reduce treatment efficacy or lead to complications [26].

There was a lot of uncertainty regarding what to do after using an EpiPen. Most participants, 222 (44.3%), were unsure about the next steps after injecting the EpiPen, and 63 (12.6%) mistakenly believed that no further action was needed after administering epinephrine. This is particularly concerning because guidelines emphasize the importance of seeking emergency medical care after using an EpiPen due to the risk of a biphasic reaction, as symptoms may reappear even after the initial treatment [27]. As a result, it is crucial for public educational campaigns to emphasize the importance of seeking follow-up care from medical professionals after using epinephrine to ensure proper treatment and monitoring.

The perception of risk associated with anaphylaxis and its potential complications also showed areas of concern. Although many participants recognized that anaphylaxis can result in severe outcomes like organ failure 191 (38%) and death 258 (51.5%), a significant number were uncertain or did not believe these complications were possible. Moreover, since anaphylaxis is a life-threatening emergency that can lead to death if not treated correctly, an unclear understanding of the complications may delay seeking appropriate care [28].

It is unclear whether anaphylaxis is hereditary or chronic. 232 (46.3%) respondents believed anaphylaxis is hereditary, and 312 (62.3%) incorrectly classified it as a chronic disease. This misunderstanding likely stems from not realizing that anaphylaxis is an acute condition typically triggered by specific exposures and not a chronic or hereditary condition [29]. For individuals with a genetic predisposition to allergic conditions, anaphylaxis can manifest as a sudden and severe allergic reaction [28].

Our study has several limitations. One of the methods used was an online survey, which limited the representation of the studied population. This was due to the impracticality of conducting an offline survey because of the social distancing mandate. To mitigate this, we increased the sample size and used a random sampling strategy across the western region of Saudi Arabia to enhance diversity and representativeness. Additionally, as the study was speculative, the findings may not fully reflect actual experiences, and self-reported responses could introduce bias. The data's generalizability is limited due to convenience sampling, making it difficult to represent all people in the western region of Saudi Arabia. Furthermore, the cross-sectional design limits the establishment of causality, while the relatively small sample size diminishes the strength of the results. Therefore, future research using prospective and longitudinal designs is recommended.

## Conclusions

The study's findings indicate that there is significant exposure to anaphylaxis in the western region of Saudi Arabia. However, there are still gaps in knowledge regarding symptoms, diagnosis, management, and prevention. The most concerning misconception involves the use of epinephrine and the correct route of administration, highlighting the need for targeted public campaigns. These measures will improve public understanding of anaphylaxis, leading to early recognition and better management of this life-threatening condition.
